# Cancer research – can the entity be bigger than the sum of its parts?

**DOI:** 10.18632/oncoscience.499

**Published:** 2020-05-01

**Authors:** Georg F. Weber

**Affiliations:** ^1^University of Cincinnati Academic Health Center, Cincinnati, Ohio, USA

**Keywords:** cancer research, policy, research funding

## Abstract

Cancer Research has benefitted from substantial expenditures by federal and nonprofit organizations. The resulting success in patient care has been uneven. Two lessons from the 20^th^ century history of science suggest infrastructural changes that can boost success. We need to better organize big science, explicitly aiming for expedient clinical translation. In parallel, resource allocation should enable investigator-initiated exploration on the basis of productivity per research dollars spent.

The War on Cancer, Personalized Medicine, Precision Medicine, Moonshot: Since the signing of the National Cancer Act of 1971, we have believed that we are finally close to ending the scourge of this disease for good, with each of these initiatives having triggered a phase of elevated confidence. Certainly, there has been progress since the dark ages before 1946, when trial and error approaches – mostly based on metal tinctures and herb extracts – dominated drug treatment. Three epochs have ensued [1]. Monotherapy with the first efficacious agents^1^ demonstrated that remission is principally achievable under chemotherapy. In 1956, Min Chiu Li, Roy Hertz and Donald B. Spencer reported their success with the methotrexate treatment of choriocarcinoma [2]. From 1965 through the close of the 20th century, clinical trials were crowded with countless permutations of combination chemotherapy, designed to spread out the horrendous adverse effects of this treatment modality, while doing the utmost to reach efficacy, thus manifesting in hard-earned remission at best. Molecular biology research into cancer flourished in parallel and – starting with the FDA approvals of rituximab (Rituxan, 1997) and imatinib mesylate (Gleevec, 2000) – ushered in the third phase. We have come a long way by pushing back the administration of non-specific DNA-damaging or anti-proliferative agents and replacing them in first line treatment with drug molecules that exploit tumor-specific changes (mutations). In addition, the targeting of tumor-host interactions has come of age with hormone treatment, anti-angiogenesis agents, and recently spectacular (albeit spotty) results with immunotherapy.

Nonetheless, the intense investment of resources and resulting high expectations have come to less than full fruition. A few years ago, a much-noticed analysis stated: “More than 40 years after the war on cancer was declared, we have spent billions fighting the good fight. The National Cancer Institute has spent some $90 billion on research and treatment during that time. Some 260 nonprofit organizations in the United States have dedicated themselves to cancer […]. Together, these 260 organizations have budgets that top $2.2 billion” [3]. Today, cancer mortality in the United States is around 163.5 per 100,000 (men and women per year, based on 2011-2015 deaths). The underlying cancer mortality rates decreased by 1.8% per year among men (2006 to 2015), 1.4% per year among women (2006 to 2015), and 1.4% per year among children (2011 to 2015) [4]. Intertwined in oncology are countless stories of either remarkable success or disturbing lack thereof.

Where have we fallen short of the noble goals? Big problems require big efforts. Prima facie, the need for coordination, collaboration, and large-scale science to aid patient care appears to have been addressed with the establishment of NCI-Designated Cancer Centers (51 Comprehensive Cancer Centers, 14 Cancer Centers, 7 Basic Laboratory Cancer Centers, all supported by NCI core grants) and the CTSA program (Clinical and Translational Science Awards, currently supporting over 50 medical research institutions). However, closer scrutiny reveals two lessons from the 20^th^ century history of science that may not have been taken into sufficient account. First, the balance between center-based, systematic, large-scale investigation and individual, investigator-driven projects has been vital for progress. Had it not been for the persistent research efforts by unique and committed minds, today’s cancer care might not have the benefits of angiogenesis inhibitors (such as bevacizumab (Avastin)) or checkpoint blockers (starting with ipilimumab (Yervoy)) among many others. The current NCI paylines for R01 grants are around 8%, about two thirds of which have been concentrated with the NCI-Designated Cancer Centers, leaving investigators at other cancer-research-active universities with a dearth of resources to pursue their mission. Nevertheless, an initiative by the NIH in recent years to balance funding disparities and relieve the on-average prevalent decline in per-dollar productivity with increasing budget has not materialized [5,6]. Secondly, the centers provide concentrations of resources and academic batting power, with limited overarching structure or research guidance. Arguably, this is in oblivion of the most monumental achievements in big science. The prototypical undertakings in this arena were the Manhattan project and the lesser known MIT Rad Lab working on radar [7,8].. Historians have suggested that radar won World War II, while the atomic bomb ended it2,3,4. Their successes have set precedents for structuring large research and development efforts that serve the public interest. Since then, stringently organized ventures have produced scientific revolutions in space exploration (moon landing, Voyager spacecraft, Hubble space telescope, international space station), physics (particle colliders), and biomedical research (the human genome). These activities were characterized by rigorously arranged overarching missions with direct application goals on tight time lines toward implementation. Neither feature is prominent in today’s cancer research infrastructure. Even though risk tolerance ought to correlate inversely with disease prognosis, only a small fraction of “promising” research has found its way from the literature to the oncology wards. At a conference, about two years back, I approached the director of a major cancer center and asked him, consecutive to his presentation on the tremendous potential offered by molecular medicine, how much of it he had implemented in patient care. The answer was telling: “We are not ready for prime time yet!” As it often goes, he wanted just one more step of progress to be completed.

For best results against cancer, the past century of research practice seems to suggest that we need to better organize large science with the explicit goal of rapid clinical translation. This structuring can be modeled from the Human Genome Project (HGP), the National Aeronautics and Space Administration (NASA), the European Organization for Nuclear Research (CERN) and similar organizations. Resource allocation needs to be judiciously balanced to also enable investigator-initiated exploration across all cancer-active academic institutes. In striving to best utilize public funds, past productivity per research dollars spent impresses as the most accurate predictor for future contributions.

## Footnotes

**Footnotes**

^1^Nitrogen mustards, originating from chemical warfare in World War I, were introduced as drugs after World War II. Independently developed, aminopterin was the first rationally designed anti-cancer agent, soon to be replaced by methotrexate.
